# Mixed-methods study to develop extensions to the SPIRIT and CONSORT statements for factorial randomised trials: the Reporting Factorial Trials (RAFT) study

**DOI:** 10.1136/bmjopen-2023-082917

**Published:** 2025-02-17

**Authors:** Sophie S Hall, Edmund Juszczak, Megan Birchenall, Diana Elbourne, Elaine Beller, An-Wen Chan, Paul Little, Alan A Montgomery, Brennan C Kahan

**Affiliations:** 1 Nottingham Clinical Trials Unit, School of Medicine, University of Nottingham, Nottingham, UK; 2 Faculty of Epidemiology and Population Health, The London School of Hygiene & Tropical Medicine, London, UK; 3 Centre for Research in Evidence Based Practice, Bond University, Gold Coast, Queensland, Australia; 4 Women's College Research Institute, University of Toronto, Toronto, Ontario, Canada; 5 Primary Care and Population Sciences, University of Southampton, Southampton, UK; 6 MRC Clinical Trials Unit at UCL, London, UK

**Keywords:** Clinical Trial, Protocols & guidelines, STATISTICS & RESEARCH METHODS

## Abstract

**Background:**

Extensions to Standard Protocol Items: Recommendations for Interventional Trials (SPIRIT) and Consolidated Standards of Reporting Trials (CONSORT) reporting recommendations specifically for factorial trials have been developed by the Reporting Factorial Trials (RAFT) study group. This article describes the processes and methods used to develop the extensions.

**Objective:**

To develop SPIRIT and CONSORT extensions for factorial trials.

**Design and participants:**

A four-phase, consensus-based approach was used: phase 1: scoping review, phase 2: Delphi survey (n=104 respondents in round 1), phase 3: consensus meeting (n=15 members) and phase 4: checklist finalisation.

**Results:**

In phase 1, the scoping review identified 31 reporting recommendations, which formed a long list of 50 concepts (19 applied to the SPIRIT extension and 31 applied to the CONSORT extension) to include in the guideline development. In phase 2, a three-round Delphi survey resulted in two new concepts being added and ended with 49 concepts (19 applied to SPIRIT and 30 applied to CONSORT) reaching consensus to remain, with only three concepts meeting the exclusion criteria. In phase 3, the concepts were further refined and translated into specific extension item wording, through an extensive review process conducted by the core RAFT team and leading trial experts, who attended a 2-day hybrid meeting. The resulting 9 SPIRIT items and 17 CONSORT items were further evaluated and developed through an iterative process in phase 4, to promote user acceptance and uptake.

**Conclusion:**

Uptake of the CONSORT and SPIRIT extensions will improve the conduct of factorial trials, as well as understanding and interpretation of such trials. By reporting on how these extensions were developed, we promote transparency of this process and share learning experiences to develop best practice when developing reporting guidelines.

**STRENGTHS AND LIMITATIONS OF THIS STUDY**

- Mixed-methods approach to develop Standard Protocol Items: Recommendations for Interventional Trials and Consolidated Standards of Reporting Trials extensions, encompassing the international literature in the field and expert opinion.
- Methods adhered to Enhancing the QUAlity and Transparency Of health Research principles for guideline development.
- International experts in the field of factorial trials and guideline reporting participated in the study.
- Alternative study methods may have produced a different set of guidelines.

## Background

 The gold standard of methodological reporting for clinical trials involves following the 33-item Standard Protocol Items: Recommendations for Interventional Trials (SPIRIT)^1^ guideline and the 25-item Consolidated Standards of Reporting Trials (CONSORT)^2 3^ guideline. These are recommendations for the minimum content of reporting trial protocols and trial results, respectively. As well as providing a checklist for best practice, the guidelines are accompanied by an Explanation and Elaboration (E&E) document, which provides further details on the items. The guidelines were originally developed to improve research transparency and reduce waste in relation to two-arm parallel group trials. Extensions have since been developed to consider the methodological nuances of specific trial designs including (but not limited to) multi-arm trials, n-of-1 trials, non-inferiority trials and pilot and feasibility trials,^4^,^8^ enabling existing items to be modified, removed or new items to be added if required.

A factorial trial design involves testing two or more interventions within the same trial.^9^ In the simplest form of a 2 × 2 design, participants are randomised to receive neither intervention, one or the other, nor both. This enables simultaneous assessment of two interventions without increasing the sample size, assuming that the interventions do not interact.^9^ Alternatively, sometimes the intent is to test for interactions between the interventions.^9^ With the ever-growing need to improve trial efficiency and offer value for money,^10 11^ factorial trials offer a potentially attractive trial design.

The use of a factorial design introduces additional methodological complexities, which are often inadequately understood and poorly reported.^12^,^15^ A review of 100 2×2 factorial trials published between January 2015 and March 2018 found a number of concerns around their design, analysis and reporting,^12^ including (1) providing no clear rationale for the factorial design, (2) a lack of reporting of interactions and (3) using a preliminary test of interaction to choose the analysis method. These limitations in conduct and reporting make it difficult to assess the validity of the trial conclusions, reducing their applicability for patient care and policy recommendations. It is essential that the rationale for using a factorial design and the assumptions made, particularly if and how an assessment of the statistical interaction was planned, are reported transparently. A simple search of the ClinicalTrials.gov registry suggests that >500 new factorial trials are registered each year, and other data suggest that 3% of all trials published in high-impact medical journals employ a factorial design.^16^ Therefore, the impact of poor reporting is potentially wide-reaching.

To facilitate improved reporting of factorial trials, the Reporting Factorial Trial (RAFT) study developed SPIRIT and CONSORT extensions^17 18^ using methods recommended for developing reporting guidelines. This article describes the consensus-based development process used to create the final checklists and to share our learning reflections and recommendations for future studies.

## Methods

The study was registered with the Enhancing the QUAlity and Transparency Of health Research (EQUATOR) network^19^ and followed their methods for developing healthcare reporting guidelines.^20^ The study protocol was published before the commencement of the research activities on an Open Science Framework.^21^

### Project launch

#### Need for the guidelines

A review of factorial trials conducted by study members highlighted key shortcomings with the reporting of factorial trials, including that the majority of published papers did not provide a rationale for using a factorial trial design, and many neglected to assess for interactions for the primary and secondary outcomes.^12^ This provided motivation for developing SPIRIT and CONSORT extensions to improve the reporting of factorial trials. A review of the EQUATOR website and published literature highlighted that no published guidelines existed to support the reporting of factorial trials.

#### Identification of research team members

The core RAFT study team was formed ensuring representation of important study-specific expertise relating to knowledge of factorial trials (BCK, AAM, EJ and PL) and the development of SPIRIT (A-WC and EJ) and CONSORT (DE, EB and EJ) guidelines. The project was supported by a Research Fellow (SSH) and a Research Co-ordinator (MB) to enable timely delivery of the project milestones. The core team met online each month, throughout the 24-month duration of the study.

#### Rationale for simultaneous development of the guidelines

The review of factorial trials^12^ highlighted deficits not only in the reporting of results but also in the design and conduct of these trials. With the goal of improving the quality of submissions to research funding bodies and governance agencies, as well as improving knowledge translation and evaluation of clinical trial results, it was decided that simultaneous development of SPIRIT and CONSORT guidelines would prove an efficient use of team resources.

#### Funding

Funding was awarded from the Medical Research Council (MRC) Methodology Research Panel to support researcher time, purchase the Delphi software, host a consensus meeting and support with dissemination. The funder played no role in the design, analysis or reporting of the study.

### Phase 1: scoping review

The objective of the scoping review was to generate a long list of concepts for consideration for inclusion in the CONSORT and SPIRIT extensions, based on reporting or methodological considerations published in the literature. Throughout the development phase, we used the term ‘concepts’ for two reasons: first, checklist ‘items’ may contain multiple reporting concepts and we wished to evaluate each of these concepts separately. Second, the actual checklist ‘item’ wording would be refined through the process reported here, and therefore, we were seeking consensus on whether the general concept was considered important as opposed to specific item wording.

#### Searches

MEDLINE search terms were developed by a research librarian with input from the team and guided by a recent similar review in this area.^12^ Given that this was a scoping review, PROSPERO (Prospective Register of Systematic Reviews) registration was not required. In line with previous reviews searching a similar topic, searches were conducted in MEDLINE from inception to May 2019.^12^ The full search strategy is presented in online supplemental material.

#### Selection

Articles were included in the scoping review if they discussed methodological or reporting issues relevant to factorial trials. One author (EB) screened titles and abstracts to remove articles that were not relevant (eg, those that reported the results of a randomised trial but not methodological issues). Another author (BCK) then further screened abstracts and full texts to assess whether articles were relevant to the SPIRIT/CONSORT extension. Reporting suggestions and methodological issues requiring additional reporting were recorded.

### Project phase 2: Delphi survey

The objective of the Delphi survey was to obtain feedback in which concepts could be removed from the long list due to perceived lower importance.

#### Participants

Sample size guidelines for Delphi studies vary from as low as 10 participants^22^ to up to 50,^23^ with other recommendations suggesting that the group characteristics are taken into consideration, ensuring there are approximately 5–10 participants representing different groups.^24^ Based on our previous experience,^25^ we aimed to recruit participants with a broad range of experience in factorial trials and guideline development (including representatives from funding bodies, journal editors and patient and public involvement (PPI)), with a target sample of n=100.

#### Recruitment strategy

We implemented two recruitment strategies concurrently. The first strategy involved sending personalised invitation letters to methodologists and trialists identified through four key sources: (1) *the RAFT team’s contacts*; (2) *relevant publications* (lead authors and methodological coauthors who had published either factorial trials, methodological work related to factorial trials and/or reporting guidelines); (3) *relevant networks* (eg, MRC-NIHR Trials Methodology Research Partnership, network of UK Clinical Research Collaboration registered Clinical Trials Units) and (4) *representatives from funding bodies* (eg, National Institute of Health Research (NIHR)) and journal editors with experience in CONSORT or SPIRIT statements. The second strategy involved wider sharing of the Delphi survey link through the RAFT study website and social media accounts associated with the author’s institutions, requesting individuals with experience and interest in factorial trials to participate in a Delphi survey. The opportunity to be entered into a prize draw to receive one of two £100 (or Great British Pound equivalent) online shopping vouchers was offered as an incentive to sign up. The prize draw was only available to participants who completed all three rounds.

#### Delphi survey process

The Delphi survey was designed and hosted online using DelphiManager software.^26^ Participants were required to read the participant information sheet, complete a brief demographic survey, specify if they wish to be entered in the prize draw and complete the consent items.

Three Delphi rounds were conducted to enable participants to suggest new items in the first round and still ensure that all items were rated at least twice. In each round, participants were requested to rate the importance of including each concept in the final set of guidelines for the SPIRIT extension and then for the CONSORT extension (presented as separate items), using a 9-point Likert scale: 1–3=not important, 4–6=important but not critical and 7–9=critical. In addition, an ‘unable to rate option’ was presented, as was space for free text to explain a given score.

After each survey round, the concept ratings were tabulated and categorised as follows:^27^

- Consensus in = ≥70% participants scoring 7–9 and<15% participants scoring 1–3.
- Consensus out = ≥70% participants scoring 1–3 and<15% of participants scoring 7–9.
- No consensus = everything else.

For each survey round, participants were invited by email (with weekly reminders sent) to complete the survey. Each round was open for 4 weeks, with a 6-week interval between subsequent survey rounds. Only those participants who completed round 1 were invited for round 2, and only participants who completed round 2 were invited for round 3.

In all survey rounds, two linked documents^28 29^ were provided on the Delphi survey and on the email informing that the survey was open. Document 1 contained keywords and definitions relevant to factorial trials to guide participants who were less familiar with factorial trial terminology. Document 2 mapped the concept for consideration in the extension guideline against the original SPIRIT and CONSORT checklist. In round 1, participants were asked to rate the concepts (n=50 concepts) and suggest new items for inclusion in the extensions if they felt appropriate.

In round 2, an additional help document was added to provide clarification for some concepts.^30^ This was developed after reviewing participants’ comments left in round 1. In round 2, participants were presented with all the items from round 1 plus two additions based on suggestions for new items (n=52 concepts). For each survey item (each concept), participants could see how their score compared with that given by other members of the survey (group ratings for each concept were displayed as percentages). Participants were required to re-rate each concept. If their new rating crossed a boundary (eg, moved from ‘important but not critical’ to ‘critical’), participants could provide a justification for changing their score if they wished to do so.

In round 3, a further document was provided to participants, which presented the reasons given by the Delphi members for changing their score where available. Participants were requested to rate the concepts that had not reached consensus and any additional items which were added after round 1.

### Project phase 3: consensus meeting

A 2-day hybrid meeting was held at the University of Nottingham. 15 participants comprised the core RAFT team (5 attended in-person and 4 online) plus six leading experts in guideline developments and publishing as well as trial design (4 in-person and 2 online). All six experts participated in the Delphi survey and had an international reputation for improving reporting standards, including authors of previous SPIRIT and CONSORT papers^31^ and editors for leading journals (BMJ and JAMA). At the meeting, concepts which had reached consensus to include were discussed in terms of their utility in being included in the extension documents and in terms of their specific wording in the final documents. Concepts which did not reach consensus were also evaluated for their importance to include in the final documents.

Before the meeting, the concepts that met the criteria for inclusion in the extension checklist after the Delphi study, were reviewed by the study team. This resulted in the concepts being condensed into specific items. These items, along with draft E&E text, were circulated to the meeting members 1 week prior.

At the meeting, a brief introduction to the RAFT study was presented to the members of the consensus meeting, summarising the methodology process and outcomes to date. Session chairs were assigned to lead and focus discussions on the concepts and E&E text. Regular breaks were held to maintain the comfort and engagement of the members. Extension texts (ie, items and E&E) were discussed first in relation to CONSORT extensions and then in relation to SPIRIT extensions. This comprised the majority of the 2-day meeting. In the latter stages of day 2, the three items not at consensus were discussed, followed by approval of the dissemination strategy. Key discussions and outcomes were recorded.

### Phase 4: checklist finalisation

Following the meeting, the core study team worked to finalise the wording of the checklist items and E&E documents. These were circulated back to the members for review through an iterative process. Three authors (AAM, BCK and EJ) pilot tested the extensions by each considering a different completed factorial randomised trial and using the checklists to briefly outline key points in the study protocol and main results publication manuscript. This process enabled the authors to reflect on the clarity of the item wording.

## Results

### Phase 1: scoping review

**Figure 1:**
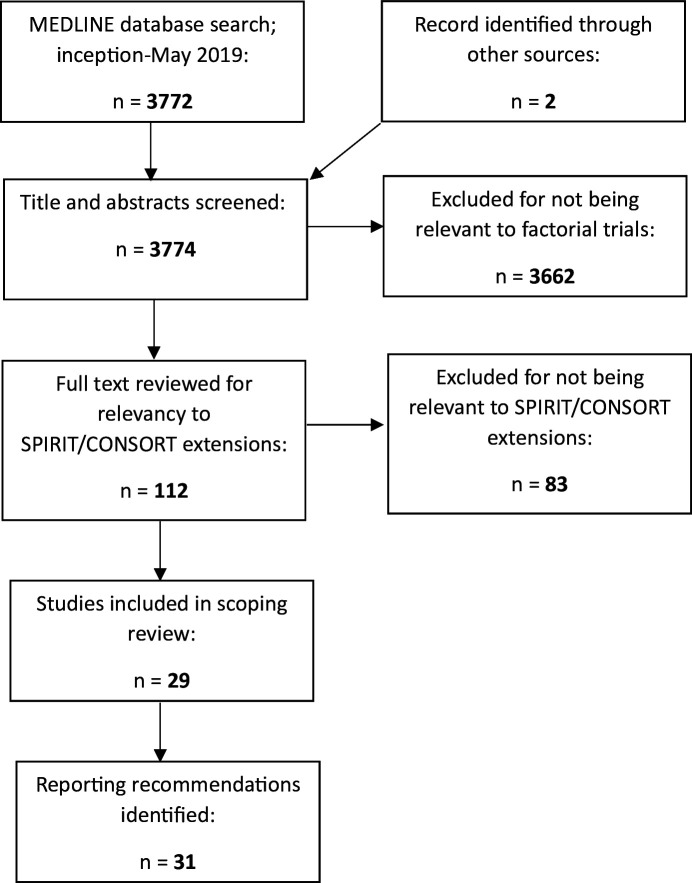
Preferred Reporting Items for Systematic Reviews and Meta-Analyses flow diagram for scoping review. CONSORT, Consolidated Standards of Reporting Trials; SPIRIT, Standard Protocol Items: Recommendations for Interventional Trials.

The records identified, included and excluded are displayed in figure 1.

A total of 3772 articles were identified from the MEDLINE search, with a further two articles included from our personal collections.^12 32^ After title/abstract screening, we were left with 112 possible articles. We excluded a further 83 articles after full-text screening, leaving 29 included papers (see Appendix 1).

A total of 31 reporting recommendations were identified from the 29 articles, 19 recommendations were applicable to the SPIRIT extension and all 31 recommendations were applicable to the development of the CONSORT extension. These reporting recommendations were termed ‘concepts’ and formed the basis for the questions in the Delphi survey.

### Phase 2: Delphi survey

Characteristics of the Delphi participants are described in table 1. The samples were predominantly statisticians. The majority had over 15 years’ experience in clinical trials, had been involved in 1–2 factorial trials and were UK based.

**Table 1:** Characteristics of the Delphi sample

Characteristic	% (n)		
	Round 1 (n=104)	Round 2 (n=95)	Round 3(n=87)
Completion rate from round 1	100	91	84 (92 from R2)
Job role*			
Statistician	57.7 (60)	60 (57)	57.5 (50)
Clinical trialist	24.0 (25)	23.2 (22)	25.3 (22)
Chief investigator	18.3 (19)	16.8 (16)	18.4 (16)
Journal editor	16.3 (17)	14.7 (14)	16.1 (14)
Trial/study manager/coordinator	6.7 (7)	6.3 (6)	5.7 (5)
Health economist	2.0 (2)	2.1 (2)	2.3 (2)
Patient and public involvement representative	2.0 (2)	2.1 (2)	2.3 (2)
Funding committee chair/member	7.7 (8)	7.4 (7)	8.0 (7)
Ethics committee chair/member	3.8 (4)	4.2 (4)	4.6 (4)
Other†	10.6 (11)	9.5 (9)	10.3 (9)
Number of factorial trials involved with			
0	19.2 (20)	18.9 (18)	20.7 (18)
1–2	49.0 (51)	49.5 (47)	47.1 (41)
3–5	21.2 (22)	20.0 (19)	20.7 (18)
6 or more	10.6 (11)	11.5 (11)	11.5 (10)
Number of years of experience in clinical trials			
Less than 1 year	1.9 (2)	2.1 (2)	2.3 (2)
1–5 years	7.7 (8)	5.3 (5)	4.6 (4)
6–14 years	32.7 (34)	35.8 (34)	36.8 (32)
15 years or more	57.5 (60)	54 (56.8)	56.3 (49)
Country of residence			
UK	63.5 (66)	62.1 (59)	60.9 (53)
Canada	11.5 (12)	11.6 (11)	12.6 (11)
USA	9.6 (10)	10.5 (10)	10.3 (9)
Europe	6.7 (7)	7.4 (7)	6.9 (6)
Australia	3.8 (4)	4.2 (4)	4.6 (4)
China	1.9 (2)	1.1 (1)	1.1 (1)
Egypt	1.0 (1)	1.1 (1)	1.1 (1)
Other	1.9 (2)	2.1 (2)	2.3 (2)

* Respondents were allowed to select more than one job role if it described their role more accurately, so the table shows their different experiences. Respondents will fit into multiple categories, so the total for each column will not equal the total sample size.

† Respondents were encouraged to choose other if none of the options provided were appropriate.

After each survey round, a report was circulated and discussed by the RAFT study group via online videoconferencing.

The results of the Delphi survey are presented in online supplemental table S1 (CONSORT) and online supplemental table S2 (SPIRIT) (see online supplemental material).

Round 1 opened on 7 January 2022, and 104 participants took part (57% of those were sent personalised letters of invitation). Of the 50 concepts which were presented for rating across the proposed SPIRIT and CONSORT extensions, 38/50 concepts (76%) met the criteria for ‘consensus in’ (SPIRIT: 15/19; CONSORT: 23/31), 0/50 concepts (0%) met the criteria for ‘consensus out’ and 12/50 concepts (24%) did not reach consensus (SPIRIT: 4/19; CONSORT: 8/31). 18 new concepts were suggested in round 1. The core RAFT team reviewed the suggestions and concluded that many were already covered by concepts that had been included in the Delphi survey or were not uniquely specific to factorial trials. One new concept was subsequently added to round 2, which applied to both SPIRIT and CONSORT and therefore resulted in the addition of two new survey items (see online supplemental tables S1 and S2).

Round 2 opened on 21 March 2022, and 95 participants took part (91% of original sample). 52 concepts were rated in round 2 (SPIRIT: 20; CONSORT: 32). The cumulative totals over the two rounds were as follows: 46/52 concepts (88%) met the criteria for ‘consensus in’ (SPIRIT: 19/20; CONSORT: 28/32), 0/52 concepts (0%) met the criteria for ‘consensus out’ and 6/52 concepts (12%) did not reach consensus (including the two new concepts added; SPIRIT: 2/20; CONSORT: 4/32).

Round 3 opened on 30 May 2022, and 87 participants took part (84% of original sample; 92% of round 2). The six concepts which did not reach consensus in round 2 were rated in round 3. In summary, the cumulative total over the three rounds were as follows: 49/52 concepts (94%) met the criteria for ‘consensus in’ (SPIRIT: 19/20; CONSORT: 30/32), 0/52 concepts (0%) met the criteria for ‘consensus out’ and 3/52 concepts (6%) did not reach consensus (SPIRIT: 1; CONSORT: 2).

The Delphi survey proved highly efficient in reaching consensus on the concepts to include in the development of SPIRIT and CONSORT extensions for factorial trials.

### Phase 3: consensus meeting

The 30 CONSORT concepts which reached consensus in the Delphi survey were discussed at the consensus meeting. Five were removed from the final checklist, either due to overlap with other concepts or because it was agreed they were not essential for the checklist (see online supplemental tables S1 and S2 footnotes). In addition, the two CONSORT concepts which did not reach consensus in the Delphi survey were discussed and removed from the final checklist for similar reasons. The 25 concepts which remained were then combined into a smaller number of checklist items for the CONSORT extension. Overall, there were 17 factorial-specific items included in the extension checklist, 16 of which were modifications to the CONSORT 2010 items and one which was a new item. The final CONSORT extension for factorial trials is available in a separate publication.^17^ Items relating to the CONSORT extension for Abstracts^31^ were not evaluated in the Delphi survey. Instead, these were chosen after the main checklist was completed in collaboration with the main author (Hopewell) of the original CONSORT for Abstracts.^31^ This approach enabled items that were agreed for inclusion in the main extension document to be considered and combined with expert topic knowledge for abstract writing, preventing the generation of an overly burdensome list given the word limits applied to abstracts.

The 19 SPIRIT concepts which reached consensus in the Delphi survey were discussed at the consensus meeting, and three were subsequently removed from the final checklist, either due to overlap with other concepts or because it was agreed they were not essential for the checklist. In addition, the one concept which did not reach consensus in the Delphi survey was discussed and removed from the final checklist for similar reasons. The 16 concepts which remained were then combined into a smaller number of checklist items for the SPIRIT extension. Overall, there were nine factorial-specific items included in the extension checklist, all of which were modifications to the SPIRIT 2013 items. The final SPIRIT extension for factorial trials is available in a separate publication.^17^ Plans for dissemination included identifying target journals for the main checklist and accompanying E&E document, highlighting the importance of publishing the methods for developing the extension checklist to ensure transparency of the process. Plans to conduct workshops/talks, including at the 2024 International Conference for Clinical Trials Methodology Conference,^33^ were also discussed with the aim of supporting researchers interested in factorial trials to conduct well-reported trials at all stages from design (ie, the protocol) to the results (ie, the manuscript).

### Phase 4: checklist finalisation

Final wording for the extension items and E&E was developed through an iterative review process between the study team and consensus meeting members. Examples of changes to the item wording include using the term ‘main comparison’ instead of ‘primary comparison’ throughout. Examples of good reporting were sourced by the study team, through rapid reviews of the literature and reviewing existing databases of factorial trials held by team members. Preliminary versions of the checklists and E&E were piloted by members of the study team. This resulted in minor modifications to the item wordings to improve reader comprehension.

## Discussion

Through a four-stage approach, we have developed SPIRIT and CONSORT extensions for factorial trials. The checklists contain the minimum important information to accurately report on the design and results of a factorial trial. The shared E&E contains further elaboration on why and how these checklists should be applied in report writing, enabling flexibility to their application in practice where applicable. As part of our evaluation of the RAFT study, we aim to share our learning reflections and recommendations for future trials (box 1).

Box 1 **Reporting Factorial Trials reflections and recommendations**

**Study development**

If extensions for Standard Protocol Items: Recommendations for Interventional Trials (SPIRIT) and Consolidated Standards of Reporting Trials (CONSORT) are required, it is resource efficient to develop them simultaneously, using one Delphi survey and one consensus meeting.

**Delphi survey**

Include key terminology and mapping documents in Delphi survey(s) to guide participants who may not be experienced in some aspects of the guideline development (eg, statistical terminology). A pilot process is useful for identifying potential areas for misunderstanding.

Consider usability and user-friendliness of Delphi software (including access to support) against the cost of licence use. There is a range of software available, which many academic institutions subscribe to, which have the functions required to run a Delphi survey.

To maximise Delphi participation, where possible, schedule the survey rounds avoiding holiday seasons and include prize draws as an incentive to complete all survey rounds.

Including larger (n>50) sample sizes in a Delphi survey has minimal logistical or workload impact.

**Consensus meeting**

Guideline development typically culminates in a consensus meeting to reach agreement on whether items which did not reach consensus in the Delphi survey should be included. However, meetings with an expert writing group, which focus on specific item wording, should also be considered to improve understanding of the final guidelines.

Hybrid meetings prevent restricting expertise to those members who cannot travel, as well as helping to reduce study costs.

**Checklist finalisation**

Identification of appropriate examples for including in the Explanation and Elaboration (E&E) represents a considerable aspect of the development of extension guidelines, but this process is often not reported. Appropriate resources should be allocated to this process to complete the extension guidelines on time.

A challenge of developing SPIRIT and CONSORT guidelines together was evident when considering publication strategies, specifically whether a shared E&E would be more appropriate than two separate ones. Key points to consider include the potential overlap between the E&E for the SPIRIT and CONSORT checklist, and conversely, the word limits imposed by many journals making a combined E&E potentially more challenging to synthesise.

To our knowledge, there are no stand-alone reports of the methodological processes used in the simultaneous development of SPIRIT and CONSORT extensions. Indeed, to date, there has been considerably more investment in developing CONSORT extensions (n=21 published on the CONSORT website, excluding translations) compared with SPIRIT (n=7 published on the SPIRIT website, excluding translations) (data correct as of March 2023).

The RAFT study has highlighted the utility in developing SPIRIT and CONSORT extensions in combination. A well-designed and reported protocol is essential not only to assess scientific, ethical, safety and design issues before trial commencement but also to facilitate evaluation of the trial on completion.^1^ Therefore, the concurrent development of both SPIRIT and CONSORT extensions promotes harmony between the two documents, encouraging consistent wording and structures to support the translation from a SPIRIT informed protocol to a CONSORT informed final report. Furthermore, developing these guidelines together proved an efficient use of resources, requiring just one panel of Delphi members and one consensus meeting.

There were several strengths to the RAFT methodology approach. First, we used a consensus-based approach, as recommended by EQUATOR^19^ for developing reporting guidelines. Our consensus threshold was clearly predefined to ensure that this was operationalised without uncertainty. Second, we involved key stakeholders from different job roles (eg, statisticians, trialists and editors), including potential end users who may have different perspectives throughout the process to help ensure that the extensions were applicable to a wide audience. Our targeted recruitment strategy resulted in over 80% of our final sample having experience of at least one factorial trial, ensuring topic-specific expertise. However, it is also advantageous that 20% had not been involved in such trials to provide independent input on the face value of the proposed items. Additionally, both our core RAFT team and our wider expert group had extensive experience in writing and publishing SPIRIT and CONSORT guidelines. This proved valuable in refining item wording, and we hope for acceptability and uptake of the guidelines. Indeed, we spent time during the 2-day meeting considering the detailed wording of specific items derived from the concepts. This approach enabled us to gain rich, nuanced information from international experts in writing (including editors of leading journals including JAMA and BMJ), which would not be possible in a Delphi survey.

Third, by conducting a scoping review of the relevant literature, we were able to consider issues which widely affect the reporting of factorial trials at an international level and then evaluate the potential importance of these issues through a Delphi survey. Indeed, this process proved highly effective, as evidenced by the majority of original items being rated as important to include and the high degree of consensus reached in the Delphi survey, with only three survey items not reaching consensus after round 3. Fourth, through our multi-strategy recruitment process, we successfully recruited experienced Delphi panel members, the majority of whom had 15 years or more experience in clinical trials. Furthermore, we were able to sustain high response rates over the three survey rounds. Reviews of the methodology of previous extensions to SPIRIT and CONSORT guidelines highlight that two-round Delphi surveys^534^,^38^ are more frequently used than three rounds;^23^ sample sizes are typically smaller than achieved in RAFT^4 5 25 36 38^ and response rates in the final round commonly vary from as low as 55–66%,^5 34 35^ with 67% being achieved in reports of a three-round Delphi survey,^25^ in contrast to the 84% achieved in round 3 of RAFT. It is possible that the inclusion of a prize draw for respondents who completed all three survey rounds contributed to these excellent response rates. By obtaining a larger sample of respondents, we were able to gain wider representation of expert opinions to reach consensus on the final extension documents. This helped to reduce content uncertainty and ensure that the guidance is broadly applicable to factorial trial designs.

Nonetheless, our study is not without limitations. It is possible that by conducting a scoping review as opposed to an extensive systematic review of the literature, some reporting recommendations were missed. This was mitigated in two ways. First, our experienced Delphi panel members were provided the opportunity to suggest additional items in round 1 of the Delphi survey. The review of these suggestions indicated that the majority were re-wordings of concepts already included. Second, members of the consensus meeting critically reflected on the clarity and comprehensiveness of the final guideline items. A second limitation of the RAFT study is that we only had two PPI participants. Participants were requested to select their main role in relation to factorial trials, and as such, it is possible that they also undertook other research-related roles. No additional feedback was requested from these PPI members regarding their inclusivity of the study design from a lay perspective. While PPI is not commonly considered in the development of reporting guidelines, patients often do their own information seeking with regards to healthcare options,^39^,^41^ and therefore, developing guidelines which promote the readability of this information to patients could be important. How best to involve PPI members who may lack the necessary experience in key research terminology is an important consideration for future research, and since conducting this study, published guidance is now available.^42^ A third limitation is that all Delphi and consensus meeting members were from high-income nations. While reports indicate that over 80% of randomised controlled trials are conducted by researchers in the top five socioeconomic countries,^43^ researchers from low- and middle-income countries are likely to have access to a different pool of resources and expertise. This may inform the judgements they make in deciding what represents critical concepts to include in reporting guidelines. Research should consider outreach activities to engage low- and middle-income countries in efforts to produce methodological guidelines.

Fourth, although a mixed-methods approach (including literature reviews and consensus-based methods), alongside substantial sample sizes, increases the generalisability and comprehensiveness of the proposed items, it remains possible that other important concepts relating to a factorial design should be reported in a study protocol and manuscript. As such, we advise that the SPIRIT and CONSORT extensions are viewed as a minimum set of items to include in the reporting process, as congruent with the original aim of the CONSORT and SPIRIT statements.^1 3^ Finally, the piloting process used represents the study’s fifth limitation. The checklist items were internally piloted by members of the immediate research team. Research to support the validity of these extension items should include piloting with less experienced researchers, evaluating individual item comprehensibility.

## Conclusion

Through a consensus-based approach, the RAFT study developed SPIRIT and CONSORT extensions to support the reporting of factorial trials. The impact of the extension documents for improving study design and reporting will be evaluated in due course, as will the applicability of the extension items in response to the proposed updates to the main SPIRIT 2013 and CONSORT 2010 guidelines (on-going at the time of writing). By reporting the methodological stages involved in the development of the RAFT guidelines, this article provides transparency for the process. Our reflections informed our recommendations for consideration in future efforts to promote best practice in the development reporting guidelines and improve the efficiency of future efforts in this area.

## supplementary material

10.1136/bmjopen-2023-082917 online supplemental file 1

10.1136/bmjopen-2023-082917 online supplemental file 2

## Data Availability

All data relevant to the study are included in the article or uploaded as supplementary information.
