# Supplementary material for: Mixed-methods study to develop extensions to the SPIRIT and CONSORT statements for factorial randomised trials: the Reporting Factorial Trials (RAFT) study
Source: BMJ Open. 2025 Feb 17;15(2):e082917. doi: 10.1136/bmjopen-2023-082917 (PMC11836851; doi:10.1136/bmjopen-2023-082917)
Supplement: online supplemental file 1 [file bmjopen-15-2-s001.docx]

*Table S1. Summary of the Consensus-based Process for Developing CONSORT Extensions for Factorial Trials*

| **Section Header** | **CONSORT 2010 No.** | **CONSORT 2010 Item** | **Extension Concept Rated in the Delphi** | **Delphi Round 1 Rating** | **Delphi Round 2 Rating** | **Delphi Round 3 Rating** |
| --- | --- | --- | --- | --- | --- | --- |
|  |  |  |  | (% rating critical / % rating not important) | | |
| Title | 1a | Identification as a randomised trial in the title | Identification as a randomised factorial trial in the title | 75.0/3.8 | 85.3/2.1 | n/a |
| Background | 2a | Scientific background and explanation of rationale | Scientific background and rationale for using a factorial design | 76.9/1.0 | 86.3/0 | n/a |
| Background | 2a | Scientific background and explanation of rationale | Justification for whether an interaction is expected or not | 67.3/2.9 | 80.0/2.1 | n/a |
| Objectives | 2b | Specific objectives or hypotheses | Specification of the research question(s) relating to the factorial design* | 81.7/2.9 | 91.6/1.1 | n/a |
| Objectives | 2b | Specific objectives or hypotheses | Clear statement of the primary comparisons involved | 96.2/0 | 100/0 | n/a |
| Trial design | 3a | Description of trial design (such as parallel, factorial) including allocation ratio | Type of factorial design (such as full or partial factorial) | 82.7/1.9 | 91.6/1.1 | n/a |
| Trial design | 3a | Description of trial design (such as parallel, factorial) including allocation ratio | Number of factors | 92.3/0 | 97.9/0 | n/a |
| Trial design | 3a | Description of trial design (such as parallel, factorial) including allocation ratio | Number of levels within each factor | 88.5/1.0 | 97.9/1.1 | n/a |
| Participants | 4a | Eligibility criteria for participants | The eligibility criteria for each factor; with any differences between the factors if applicable | 81.7/1.9 | 92.6/0 | n/a |
| Sample size | 7a | How sample size was determined | Planned sample size with details of how it was determined for each primary comparison | 89.4/1.0 | 93.7/1.1 | n/a |
| Sample size | 7a | How sample size was determined | Whether an interaction was assumed in the sample size calculation | 77.9/2.9 | 88.4/2.1 | n/a |
| Interim analysis and stopping guidelines | 7b | When applicable, explanation of any interim analyses and stopping guidelines | Stopping guidelines for each factor; with any differences between the factors if appropriate | 64.4/2.9 | 73.7/0 | n/a |
| Sequence generation | 8b | Type of randomisation; details of any restriction (such as blocking and block size) | Time-point of randomisation for each factor | 61.5/1.0 | 76.8/0 | n/a |
| Statistical methods | 12a | Statistical methods used to compare groups for primary and secondary outcomes | Description of estimand(s) for each primary and secondary outcome (treatment comparison; population; outcome definition; population-level summary; handling of intercurrent events)* | 75.0/5.8 | 84.2/3.2 | n/a |
| Statistical methods | 12a | Statistical methods used to compare groups for primary and secondary outcomes | Primary approach to statistical analysis (such as factorial; multi-arm) used to compare groups for primary and secondary outcomes; and details on how this approach will be chosen | 85.6/1.0 | 97.9/0 | n/a |
| Statistical methods | 12a | Statistical methods used to compare groups for primary and secondary outcomes | How the other factor(s) will be handled during analysis | 69.2/3.8 | 83.2/2.1 | n/a |
| Statistical methods | 12a | Statistical methods used to compare groups for primary and secondary outcomes | Whether any adjustments for multiplicity will be applied and method used* | 70.2/2.9 | 85.3/2.1 | n/a |
| Statistical methods | 12b | Methods for additional analyses, such as subgroup analyses and adjusted analyses | Method(s) used to evaluate evidence of statistical interactions | 73.1/1.9 | 86.3/1.1 | n/a |
| Statistical methods | 12b | Methods for additional analyses, such as subgroup analyses and adjusted analyses | Likely impact of potential interactions on interpretation* | 65.4/4.8 | 68.4/3.2 | 71.3/1.1 |
| Participant flow | 13a | For each group, the numbers of participants who were randomly assigned, received intended treatment, and were analysed for the primary outcome | For each primary comparison; the numbers of participants who were randomly assigned; received intended treatment; and were analysed for the primary outcome | 95.2/0 | 100/0 | n/a |
| Participant flow | 13b | For each group, losses and exclusions after randomisation, together with reasons | For each primary comparison; losses and exclusions after randomisation; together with reasons | 94.2/0 | 100/0 | n/a |
| Recruitment | 14a | Dates defining the periods of recruitment and follow-up | Dates defining the periods of recruitment and follow-up; if different across factors; describe reason(s) for the differences and any statistical implications | 74.0/1.0 | 87.4/1.1 | n/a |
| Baseline data | 15 | A table showing baseline demographic and clinical characteristics for each group | A table showing baseline demographic and clinical characteristics for each primary comparison | 85.6/0 | 91.6/0 | n/a |
| Numbers analysed | 16 | For each group, number of participants (denominator) included in each analysis and whether the analysis was by original assigned groups | For each primary comparison; the number of participants (denominator) included in each analysis and whether the analysis was by original assigned groups | 91.3/0 | 95.8/0 | n/a |
| Outcomes and estimation | 17a | For each primary and secondary outcome, results for each group, and the estimated effect size and its precision (such as 95% confidence interval) | For each primary and secondary outcome; results for each primary comparison; the estimated effect size and its precision (such as 95% confidence interval) | 96.2/0 | 100/0 | n/a |
| Outcomes and estimation | 17a | For each primary and secondary outcome, results for each group, and the estimated effect size and its precision (such as 95% confidence interval) | For each primary and secondary outcome; the estimated interaction effect and its precision | 77.9/1.9 | 84.2/3.2 | n/a |
| Ancillary analysis | 18 | Results of any other analyses performed, including subgroup analyses and adjusted analyses, distinguishing pre-specified from exploratory | Outcome data (including primary and secondary outcomes; harms; and adherence) presented by multi-arm group | 70.2/2.9 | 87.4/2.1 | n/a |
| Harms | 19 | All important harms or unintended effects in each group | All important harms or unintended effects in each primary comparison | 85.6/0 | 100/0 | n/a |
| Limitations | 20 | Trial limitations, addressing sources of potential bias, imprecision, and, if relevant, multiplicity of analyses | Influence of potential interactions | 64.4/2.9 | 84.2/1.1 | n/a |
| Limitations | 20 | Trial limitations, addressing sources of potential bias, imprecision, and, if relevant, multiplicity of analyses | Whether adherence to intervention might have been affected by inclusion of other factors | 48.1/8.7 | 51.6/6.3 | 37.9/4.6 |
| Limitations | 20 | Trial limitations, addressing sources of potential bias, imprecision, and, if relevant, multiplicity of analyses | Effect of multiplicity of analyses (if relevant) | 49.0/4.8 | 51.6/2.1 | 48.3/2.3 |
| n/a | n/a | n/a, item added in response to round 1 feedback | Whether effect(s) of the other intervention(s) on the assumed control group event rate have been accounted for when calculating sample size (applies to binary primary outcome)* | Added after round 1 | 62.1/6.3 | 74.7/4.6 |

*Removed due to over-lap with other items (2b), lack of specific relevance to factorial trials (12a x2 and 12b), or because it does not broadly apply to all types of factorial trial designs (new item, no corresponding number)

*Table S2. Summary of the Consensus-based Process for Developing SPIRIT Extensions for Factorial Trials*

| **Section Header** | **SPIRIT 2013 No.** | **Extension No** | **SPIRIT 2013 Item** | **Extension Concept Rated in the Delphi** | **Delphi Round 1 Rating** | **Delphi Round 2 Rating** | **Delphi Round 3 Rating** |
| --- | --- | --- | --- | --- | --- | --- | --- |
|  |  |  |  |  | % rating critical / % rating not important | | |
| Title | 1 |  | Descriptive title identifying the study design, population, interventions, and, if applicable, trial acronym | Identification as a randomised factorial trial in the title | 73.1/3.8 | 84.2/2.1 | n/a |
| Background and rationale | 6a & 6b |  | Description of research question and justification for undertaking the trial, including summary of relevant studies (published and unpublished) examining benefits and harms for each intervention | Scientific background and rationale for using a factorial design | 83.7/0 | 91.6/0 | n/a |
| Background and rationale | 6a & 6b |  | Description of research question and justification for undertaking the trial, including summary of relevant studies (published and unpublished) examining benefits and harms for each intervention | Justification for whether an interaction was expected or not | 72.1/1.0 | 86.3/1.1 | n/a |
| Objectives | 7 |  | Specific objectives or hypotheses | Specification of the research question(s) relating to the factorial design* | 84.6/2.9 | 93.7/1.1 | n/a |
| Objectives | 7 |  | Specific objectives or hypotheses | Clear statement of the primary comparisons involved | 95.2/0 | 100/0 | n/a |
| Trial design | 8 |  | Description of trial design including type of trial (e.g., parallel group, crossover, factorial, single group), allocation ratio, and framework (e.g., superiority, equivalence, noninferiority, exploratory) | Type of factorial design (such as a full or partial factorial) | 86.5/1.9 | 94.7/1.1 | n/a |
| Trial design | 8 |  | Description of trial design including type of trial (e.g., parallel group, crossover, factorial, single group), allocation ratio, and framework (e.g., superiority, equivalence, noninferiority, exploratory) | Number of factors | 92.3/0 | 97.9/0 | n/a |
| Trial design | 8 |  | Description of trial design including type of trial (e.g., parallel group, crossover, factorial, single group), allocation ratio, and framework (e.g., superiority, equivalence, noninferiority, exploratory) | Number of levels within each factor | 87.5/1.0 | 96.8/1.1 | n/a |
| Eligibility Criteria | 10 |  | Inclusion and exclusion criteria for participants. If applicable, eligibility criteria for study centres and individuals who will perform the interventions (e.g., surgeons, psychotherapists) | The eligibility criteria for each factor, with any differences between the factors if applicable | 86.5/1.0 | 94.7/0 | n/a |
| Sample Size | 14 |  | Estimated number of participants needed to achieve study objectives and how it was determined, including clinical and statistical assumptions supporting any sample size calculations | Planned sample size with details of how it was determined for each primary comparison | 96.2/0 | 98.9/0 | n/a |
| Sample Size | 14 |  | Estimated number of participants needed to achieve study objectives and how it was determined, including clinical and statistical assumptions supporting any sample size calculations | Whether an interaction was assumed in the sample size calculation | 80.8/1.9 | 90.5/1.1 | n/a |
| Sequence generation | 16a |  | Method of generating the allocation sequence (e.g., computer generated random numbers), and list of any factors for stratification. To reduce predictability of a random sequence, details of any planned restriction (e.g., blocking) should be provided in a separate document that is unavailable to those who enrol participants or assign interventions | Time-point of randomisation for each factor | 70.2/1.0 | 84.2/0 | n/a |
| Statistical methods | 20a |  | Statistical methods for analysing primary and secondary outcomes. Reference to where other details of the statistical analysis plan can be found, if not in the protocol | Description of estimand(s) for each primary and secondary outcome (treatment comparison, population, outcome definition, population-level summary, handling of intercurrent events)* | 70.2/4.8 | 81.1/2.1 | n/a |
| Statistical methods | 20a |  | Statistical methods for analysing primary and secondary outcomes. Reference to where other details of the statistical analysis plan can be found, if not in the protocol | Primary approach to statistical analysis (such as factorial, multi-arm) used to compare groups for primary and secondary outcomes, and details on how this approach will be chosen | 83.7/1.0 | 95.8/0 | n/a |
| Statistical methods | 20a |  | Statistical methods for analysing primary and secondary outcomes. Reference to where other details of the statistical analysis plan can be found, if not in the protocol | How the other factor(s) will be handled during analysis | 61.5/4.8 | 72.6/1.1 | n/a |
| Statistical methods | 20a |  | Statistical methods for analysing primary and secondary outcomes. Reference to where other details of the statistical analysis plan can be found, if not in the protocol | Whether any adjustments for multiplicity will be applied and method used* | 64.4/4.8 | 80.0/3.2 | n/a |
| Statistical methods | 20b |  | Methods for any additional analyses (e.g., subgroup and adjusted analyses) | Method(s) used to evaluate evidence of statistical interactions | 66.3/4.8 | 76.8/2.1 | n/a |
| Statistical methods | 20b |  | Methods for any additional analyses (e.g., subgroup and adjusted analyses) | Likely impact of potential interactions on interpretation | 52.9/8.7 | 47.4/5.3 | 36.8/3.4 |
| Data monitoring | 21b |  | Description of any interim analyses and stopping guidelines, including who will have access to these interim results and make the final decision to terminate the trial | Stopping guidelines for each factor, with any differences between the factors if appropriate | 72.1/1.9 | 84.2/0 | n/a |
| n/a | n/a |  | n/a, item added after round 1 | Whether effect(s) of the other intervention(s) on the assumed control group event rate have been accounted for when calculating sample size (applies to binary primary outcome)* | Added after round 1 | 65.3/4.2 | 80.5/3.4 |

*Removed due to over-lap with other items (7), lack of specific relevance to factorial trials (20a x 2), or because it does not broadly apply to all types of factorial trial designs (new item, no corresponding number.
