# Supplementary material for: Mixed-methods study to develop extensions to the SPIRIT and CONSORT statements for factorial randomised trials: the Reporting Factorial Trials (RAFT) study
Source: BMJ Open. 2025 Feb 17;15(2):e082917. doi: 10.1136/bmjopen-2023-082917 (PMC11836851; doi:10.1136/bmjopen-2023-082917)
Supplement: online supplemental file 2 [file bmjopen-15-2-s002.docx]

**SUPPLEMENTARY MATERIAL**

**Study Protocol**

<https://osf.io/e3n5r/files/osfstorage/61a8cd100db659030d835575>

**Search Terms**

The search terms used in the scoping review were:

(((Factorial OR "2 x 2" OR 2x2 OR "Two by two" OR "Four arm" OR "Four-arm")) AND ("Randomized Controlled Trials as Topic"[Mesh] OR Trial OR Trials)) AND ("Research Design"[Mesh] OR "Research Design" OR "Research Designs" OR "Factorial Design" OR "Factorial Designs" OR "Research Techniques" OR "Research Technique" OR "Experimental Design" OR "Experimental Designs" OR "Research Methodology" OR "Research methods"). The search was supplemented with searches of our personal collections of pre-print articles.

**Papers Included in the Scoping Review**

| 1 | Allore, H. G. & T. E. Murphy (2008). An examination of effect estimation in factorial and standardly-tailored designs. *Clinical Trials 5*(2): 121-130. |
| --- | --- |
| 2 | Baker, T. B., et al. (2017). Implementing Clinical Research Using Factorial Designs: A Primer. *Behavioural Therapy 48*(4): 567-580. |
| 3 | Bria, E., et al. (2006). Factorial design for randomized clinical trials. *Annals of Oncology 17*(10): 1607-1608. |
| 4 | Brittain, E. & J. Wittes (1989). Factorial designs in clinical trials: the effects of non-compliance and subadditivity. *Statistics in Medicine* *8*(2): 161-171. |
| 5 | Byar, D. P. (1989). Some statistical considerations for design of cancer prevention trials. *Preventive Medicine* *18*(5): 688-699. |
| 6 | Byar, D. P., et al. (1993). Incomplete factorial designs for randomized clinical trials. *Statistics in Medicine 12*(17): 1629-1641. |
| 7 | Byth, K. & V. Gebski (2004). Factorial designs: a graphical aid for choosing study designs accounting for interaction. *Clinical Trials 1*(3): 315-325. |
| 8 | Cairns, J., et al. (1991). Issues in the early termination of the aspirin component of the Physicians' Health Study. Data Monitoring Board of the Physicians' Health Study. *Annals of Epidemiology 1*(5): 395-405. |
| 9 | Collins, L. M., et al. (2014). Factorial experiments: efficient tools for evaluation of intervention components. *American Journal of Preventive Medicine 47*(4): 498-504. |
| 10 | Crespi, C. M. (2016). Improved Designs for Cluster Randomized Trials*. Annual Review of Public Health 37*: 1-16. |
| 11 | Curran, D., et al. (1999). Sample size estimation in phase III cancer clinical trials. *European Journal of Surgical Oncology 25*(3): 244-250. |
| 12 | Dakin, H. & A. Gray (2017). Economic evaluation of factorial randomised controlled trials: challenges, methods and recommendations. *Statistics in Medicine 36*(18): 2814-2830. |
| 13 | Dakin, H. A., et al. (2018). Partial factorial trials: comparing methods for statistical analysis and economic evaluation. *Trials 19*(1): 442. |
| 14 | Foley, R. N. (2009). Analysis of randomized controlled clinical trials. *Methods in Molecular Biology 473*: 113-126. |
| 15 | Freidlin, B. & E. L. Korn (2017). Two-by-Two Factorial Cancer Treatment Trials: Is Sufficient Attention Being Paid to Possible Interactions? *Journal of the National Cancer Institute 109*(9). |
| 16 | Green, S., et al. (2002). Factorial design considerations. *Journal of Clinical Oncology 20*(16): 3424-3430. |
| 17 | Green, S. B. (2000). Hypothesis testing in clinical trials. *Hematology Oncology Clinics of North America 14*(4): 785-795, vii-viii. |
| 18 | Kahan, B. C. (2013). Bias in randomised factorial trials. *Statistics in Medicine 32*(26): 4540-4549. |
| 19 | Korn, E. L. & B. Freidlin (2016). Non-factorial analyses of two-by-two factorial trial designs. *Clinical Trials 13*: 651-659. |
| 20 | Larntz, K., et al. (1996). Data analysis issues for protocols with overlapping enrollment. *Statistics in Medicine 15*(21-22): 2445-2453. |
| 21 | Lubsen, J. & S. J. Pocock (1994). Factorial trials in cardiology: pros and cons. *European Heart Journal 15*(5): 585-588. |
| 22 | McAlister, F. A., et al. (2003). Analysis and reporting of factorial trials: a systematic review. *JAMA 289*(19): 2545-2553. |
| 23 | McClure, L. A., et al. (2013). Monitoring futility in a two-by-two factorial design: the SPS3 experience. *Clinical Trials 10*(2): 250-256. |
| 24 | Mdege, N. D., et al. (2014). The 2 x 2 cluster randomized controlled factorial trial design is mainly used for efficiency and to explore intervention interactions: a systematic review. *Journal of Clinical Epidemiology 67*(10): 1083-1092. |
| 25 | Montgomery, A. A., et al. (2011). Reporting of factorial trials of complex interventions in community settings: a systematic review. *Trials 12*: 179. |
| 26 | Montgomery, A. A., et al. (2003). Design, analysis and presentation of factorial randomised controlled trials. *BMC Medical Research Methodology 3*: 26. |
| 27 | Pocock, S. J., et al. (2015). Challenging Issues in Clinical Trial Design: Part 4 of a 4-Part Series on Statistics for Clinical Trials. *Journal of the American College of Cardiology 66*(25): 2886-2898. |
| 28 | Kahan, B. C. et al. (2020). Reporting of randomized factorial trials was frequently inadequate. *Journal of Clinical Epidemiology* *117*: 52-59. |
| 29 | Juszczak, E., et al. (2019). Reporting of multi-arm parallel-group randomized trials: extension of the CONSORT 2010 statement. *JAMA* *321*(16): 1610-1620. |
