# Supplementary material for: Mixed-methods study to develop extensions to the SPIRIT and CONSORT statements for factorial randomised trials: the Reporting Factorial Trials (RAFT) study
Source: BMJ Open. 2025 Feb 17;15(2):e082917. doi: 10.1136/bmjopen-2023-082917 (PMC11836851; doi:10.1136/bmjopen-2023-082917)
Supplement: Supplementary file 3 [file bmjopen-2023-082917.reviewer_comments.pdf]

# PEER REVIEW HISTORY

BMJ Open publishes all reviews undertaken for accepted manuscripts. Reviewers are asked to complete a checklist review form and are provided with free text boxes to elaborate on their assessment. These free text comments are reproduced below.

## ARTICLE DETAILS

### Title (Provisional)

A mixed methods study to develop extensions to the SPIRIT and CONSORT statements for factorial randomised trials: the Reporting Factorial Trials (RAFT) Study

### Authors

Hall, Sophie; Juszczak, Edmund; Birchenall, Megan; Elbourne, Diana; Beller, Elaine; Chan, An-Wen; Little, Paul; Montgomery, Alan; Kahan, Brennan C

---

## VERSION 1 - REVIEW

---

| | |
|---|---|
| **Reviewer** | **1** |
| **Name** | **Huot, Laure** |
| **Affiliation** | **Hospices Civils de Lyon, Pole Sante Publique** |
| **Date** | **11-Mar-2024** |
| **COI** | **None** |

---

Thank you for the opportunity to revise this work. This well-written publication presents the method used to develop extensions to the CONSORT and SPIRIT checklist for factorial clinical trials, which have been already published. It provides interesting recommendations for teams wishing to produce other extensions to such checklists. Here are some suggestions and comments for improving the manuscript.

ABSTRACT

Some revisions should be made in the abstract.

The RAFT study should be mentioned and defined in the background paragraph for better understanding.

To be consistent with the manuscript, it should be mentioned “leading experts” rather than “wider RAFT members” in the phase 3 results.

The conclusion should focus on the work presented in the manuscript (i.e. feedback and recommendations for the method of developing extensions to the reporting guidelines), rather than conclusion related to the extension of SPIRIT and CONSORT themselves, which concern other published articles.

METHODS

Concerning recruitment strategy, it is not clear whether the two stages took place in parallel, or whether the number of people recruited in the 1st stage was not sufficient and led the team to expand the group in the 2nd stage. Was the prize draw offered to all participants, or only to the 2nd stage?

We understand that the people targeted in the first phase are mainly people from the UK (which could explain some of the results in terms of respondent characteristics). Some details could be added about the means used to identify targeted people in the 2nd stage; for example, which social media accounts were used (only professionals?).

In table 1, the "other" country of residence could perhaps be detailed (only 2 people concerned).

Line 21-22: the sentence seems to be truncated ("the dissemination strategy").

RESULTS

There is an error in Figure 1: it should read "3772" articles retrieved from the Meline database search. Furthermore, the top right-hand box should be linked to the "title and abstracts screened" box rather than to the "Medline database search" box.

DISCUSSION

Box 1 is very interesting for future development of checklist extensions.

I have few questions regarding the Delphi process, that could be addressed in the discussion section. Did the authors encountered any logistical difficulties in including such a large number of participants? With regard to the representativeness of participants from different countries, how did the recruitment method allow researchers from outside the UK and other Western countries to be targeted? What was the added value of including so many participants in the Delphi results, compared to other works? How do the authors explain the fact that almost all the concepts were finally retained in the final results of the Delphi process?

---

**Reviewer** 2

**Name** Jamrozik, Euzebiusz

**Affiliation** University of Oxford, Ethox and the Wellcome Centre for Ethics and Humanities

**Date** 02-Apr-2024

**COI** nil

---

This is an excellent paper that makes progress on an important topic. I have only minor comments as suggestions for areas where it might have been interesting to hear more detail:

- Some concepts were removed because "it was agreed they were not essential for the checklist."; I could not find key reasons for these decisions in the manuscript, and in some cases it might be useful to know why certain concepts were removed

- The authors note that 80% of their respondents were involved in factorial trials, which was a noted advantage of their recruitment strategy, but it might also be advantageous to have had the 20% who had not been involved in such trials to provide independent input

- "all Delphi and consensus meeting members were from developed westernised countries.": was this an intentional part of study design? what proportion of factorial trials occur in such countries? and could the word "westernised' be removed or clarified?

---


| | |
|---|---|
| **Reviewer** | **3** |
| **Name** | **Kawade, Anand** |
| **Affiliation** | **King Edward Memorial Hospital, Vadu Rural Heath Progrm** |
| **Date** | **06-Sep-2024** |
| **COI** | **I declared that I have no competing interest.** |


---

At the outset I would like to congratulate the authors for this great effort. As the science of clinical trials progresses, more and more complex studies are inevitable. Factorial design is one of them. However, there are critical challenges with factorial trial with respect to design, analysis and reporting. due to lack of clear guidance. RAFT team having huge experience had taken up the challenge and developed the SPIRIT and CONSORT extension appropriate for factorial trials.

The manuscript is very well written and it gives complete clarity to reader. I have no comments specific to any of the section but to have few suggestions:

1]Interaction scores for factors:

This score is based on known or presumed interactions between factors.

1. Interactions could be negative or positive (+/_)

2. Based on previous knowledge, we could assess the interactions as

No interaction==0, Some interaction ==1 and significant interaction==2

Thus, we can make a metrics assessing interaction scores for primary and secondary outcomes as below:

Outcome Interaction score

Positive Negative

Primary

Secondary

Total score

Example:

Suppose we are testing two factors A & B

For primary outcome they have significant positive interaction (Additive effect) while for secondary outcome there is some negative interaction. So, the total score could be [2+(-1) = 1]. Thus, we can go ahead and keep this interaction in mind during analysis. If total score is negative, then you may refrain.

2] While conducting factorial trial in community, the compliance, contamination and flexibility of interaction (especially in case of behavioral intervention) could be very dynamic. These should be considered while reporting.

3) Word limitation by journal is a big challenge. Since the details are not required by every reader, journal can ask to submit this explanation /information as supplementary documents for reader who is interested in nuances of study.

CONGRATULATIONS again.

---

| | |
|---|---|
| **Reviewer** | **4** |
| **Name** | **Elsman, Ellen** |
| **Affiliation** | **Amsterdam UMC Location VUmc, Epidemiology & Data Science** |
| **Date** | **20-Nov-2024** |
| **COI** | **I have no competing interest,** |

---

This manuscript explains the process and results to develop an extension of the SPIRIT and CONSORT reporting guidelines for factorial design trials. It consisted of multiple steps, including literature review, Delphi surveys, and a consensus meeting, as is advised for reporting guideline development projects. I enjoyed reading this paper, but identified multiple aspects that need further clarification or reflection.

My main concern centers around the focus on aspects. A rationale is provided as to why the focus was on concepts, instead of items. However, because the focus was on concepts, there was no testing about the clarity of item wording. In my opinion, this is a major limitation, because a Delphi study can give information on the comprehensiveness, relevance and comprehensibility of reporting items (very similar to a content validity study). Also, from my experience with reporting guideline development through Delphi studies, there is often high consensus on the relevance of items, but less so on the wording of reporting items, and the

subsequent Delphi rounds really help to improve wording. The choice to focus on concepts instead of reporting items prevented this from happening. I therefore wonder if there will be any subsequent evaluation of item wording and how improvements or revisions are planned?

Another concern is about the pilot testing. It seems that pilot testing was conducted with people who were very involved in the project and thus are very familiar with the meaning of reporting items. Wouldn't it be better to pilot test with people new to the reporting items, so that more information about the clarity of items can be obtained? Also, pilot testing is very briefly described, and would benefit from more information. For example, how was the checklist pilot tested? How many articles were used? What was the feedback? Was the E&E document pilot tested as well?

PPI in reporting guideline development is relatively new. There were 2 PPI representatives involved in the Delphi study. Was there role solely PPI representative, or did they had other job roles as well (e.g., research-focused roles)? If not, were you able to collect experiences from them, and were there any measures in place to make sure they were able to understand the process and content? In the discussion, you already reflect on the limitation that only two PPI members were involved, and this might be a good place to refer to guidelines on PPI in reporting guideline development:

"A blueprint for patient and public involvement in the development of a reporting guideline for systematic reviews of outcome measurement instruments: PRISMA-COSMIN for OMIs 2024." Research involvement and engagement 10.1 (2024): 33.

It is stated that factorial trial designs are often not reported completely. Which aspects in particular are often reported incompletely? Could you give some examples? This might help the reader better understand the reporting issues that contribute to limited applicability.

Please provide more information on the 6 additional consensus meeting participants. Who were they and how were they selected? Did they participate in the Delphi study as well? And where they all joining online?

Items relating to the abstract were not evaluated in the Delphi survey but were chosen after completion of the main checklist. Why was this approach followed, how were items chosen and by whom?

What strategies for dissemination of the RAFT guidelines are in place?

Empirical studies quantify uncertainty a number of ways, but it is relevant to any scientific endeavor. How do you characterize uncertainty in the process of developing the RAFT guidelines? Obviously if you did the whole thing again (w/ or w/o different participants) you would expect some variability in the outcome. How is this characterized for this particular study?

Minor issues:

Spell out RAFT in the abstract

"Funding was awarded from the Medical Research Council (MRC) Methodology Research Panel to support researcher time, purchase the Delphi software, host a consensus meeting. and support with dissemination." > please remove the period in the middle of the sentence.

"First, our experienced Delphi panel members were provided the opportunity to suggest additional items in round of the Delphi survey." > should read round 1?

"A second limitation of the RAFT study is that we only had two from Patient and Public Involvement (PPI) participants." > please deleted from

---

## VERSION 1 - AUTHOR RESPONSE

**Reviewer: 1 Dr. Laure Huot, Hospices Civils de Lyon, Université Lyon 1**

**ABSTRACT**

1. The RAFT study should be mentioned and defined in the background paragraph for better understanding.

- Text has been added to background of the Abstract:

"Extensions to SPIRIT and CONSORT reporting recommendations specifically for factorial trials have been developed by the Reporting Factorial Trials (RAFT) study group. This article describes the processes and methods used to develop the extensions."

2. To be consistent with the manuscript, it should be mentioned "leading experts" rather than "wider RAFT members" in the phase 3 results.

- Text has been amended as suggested.

3. The conclusion should focus on the work presented in the manuscript (i.e. feedback and recommendations for the method of developing extensions to the reporting guidelines), rather than conclusion related to the extension of SPIRIT and CONSORT themselves, which concern other published articles.

- The conclusion in the Abstract has been revised as follows:

"Uptake of the CONSORT and SPIRIT extensions will improve conduct of factorial trials, as well as understanding and interpretation of such trials. By reporting on how these extensions were developed, we promote transparency of this process and share learning experiences to develop best practice when developing reporting guidelines."

**METHODS**

1. Concerning recruitment strategy, it is not clear whether the two stages took place in parallel, or whether the number of people recruited in the 1st stage was not sufficient and led the

team to expand the group in the 2nd stage. Was the prize draw offered to all participants, or only to the 2nd stage?

- We have revised the Recruitment strategy as follows:

"We implemented two recruitment strategies concurrently. The first strategy involved sending personalised invitation letters to methodologists and trialists identified through four key sources:.."

"The second strategy involved wider sharing of the Delphi survey link through the RAFT study website and social media accounts associated with the author's institutions, requesting individuals with experience and interest in factorial trials to participate in a Delphi survey."

"The opportunity to be entered into a prize draw to receive one of two £100 (or Great British Pound equivalent) online shopping vouchers was offered as an incentive to sign up. The prize draw was only available to participants who completed all three rounds."

2. We understand that the people targeted in the first phase are mainly people from the UK (which could explain some of the results in terms of respondent characteristics). Some details could be added about the means used to identify targeted people in the 2nd stage; for example, which social media accounts were used (only professionals?).

- We have added further detail (please see text in response to the comment above).

3. In table 1, the "other" country of residence could perhaps be detailed (only 2 people concerned).
   Line 21-22: the sentence seems to be truncated ("the dissemination strategy").

- It is not possible to state information relating to country of residence, as the response item was 'other' with no further information available.
- The wording has been revised as follows:

"In the latter stages of day 2, the three items not at consensus were discussed, followed by approval of the dissemination strategy."

**RESULTS**

There is an error in Figure 1: it should read "3,772" articles retrieved from the Meline database search. Furthermore, the top right-hand box should be linked to the "title and abstracts screened" box, rather than to the "Medline database search" box.

- The figure has been updated accordingly, correcting both issues.

**DISCUSSION**

1. Did the authors encountered any logistical difficulties in including such a large number of participants?

- No logistical difficulties were encountered - this has been added as reflection point in Box 1, as it may provide useful information for other researchers planning similar studies.

2. With regard to the representativeness of participants from different countries, how did the recruitment method allow researchers from outside the UK and other Western countries to be targeted?

➢ We recognise that our recruitment method did not specifically target low- and-middle income countries, as such we reflect upon this in the discussion:

"A third limitation is that all Delphi and consensus meeting members were from high income nations. Whilst reports indicate that over 80% of randomised controlled trials are conducted by researchers in the top five socio-economic countries (43), researchers from low- and middle income countries are likely to have access to a different pool of resources and expertise. This may inform the judgements they make in deciding what represent critical concepts to include in reporting guidelines. Research should consider out-reach activities to engage low- and middle income countries in efforts to produce methodological guidelines."

3. What was the added value of including so many participants in the Delphi results, compared to other works?

➢ We have reflected on this in our discussion:

"By obtaining a larger sample of respondents we were able to gain wider representation of expert opinion to reach consensus on the final extension documents. This helped to reduce content uncertainty and ensure the guidance is broadly applicable to factorial trial designs."

4. How do the authors explain the fact that almost all the concepts were finally retained in the final results of the Delphi process?

➢ We have now included this as a discussion point:

"Thirdly, by conducting a scoping review of the relevant literature we were able to consider issues which widely affect the reporting of factorial trials at an international level, and then evaluate the potential importance of these issues through a Delphi survey. Indeed, this process proved highly effective as evidenced by the majority of original items being rated as important to include and the high degree of consensus reached in the Delphi survey, with only three survey items not reaching consensus after round 3."

**<u>Reviewer: 2: Dr. Euzebiusz Jamrozik, University of Oxford, University of Melbourne</u>**

1. Some concepts were removed because "it was agreed they were not essential for the checklist."; I could not find key reasons for these decisions in the manuscript, and in some cases it might be useful to know why certain concepts were removed.

➢ We have clarified this by marking these items in Table 2 and Table 3, with a footnote added to explain why they were removed. We have also referred readers to this in the main text:

"The 30 CONSORT concepts which reached consensus in the Delphi survey were discussed at the consensus meeting. Five were removed from the final checklist, either due to overlap with other concepts, or because it was agreed they were not essential for the checklist (see Table 2 and 3 footnotes)."

2. The authors note that 80% of their respondents were involved in factorial trials, which was a noted advantage of their recruitment strategy, but it might also be advantageous to have had the 20% who had not been involved in such trials to provide independent input.

- We have included this in our discussion:

"Our targeted recruitment strategy ensured that over 80% of our final sample had been involved in at least one factorial trial, ensuring topic-specific expertise. However, it is also advantageous that 20% had not been involved in such trials, to provide independent input on the face value of the proposed items."

3. "all Delphi and consensus meeting members were from developed westernised countries.": was this an intentional part of study design? what proportion of factorial trials occur in such countries? and could the word "westernised' be removed or clarified.

- We have changed the term Westernised. Information relating to the proportion of factorial trials conducted globally is lacking. However, we make an important reflection of the proportion of clinical trials conducted, generally, in high- and low-income countries.

"Whilst reports indicate that over 80% of randomised controlled trials are conducted by researchers in the top five socio-economic countries (40), researchers from low and middle income countries are likely to have access to a different pool of resources and expertise. This may inform the judgements they make in deciding what represent critical concepts to include in reporting guidelines."

**Reviewer: 3 Dr. Anand Kawade, King Edward Memorial Hospital, King Edward Memorial Hospital Pune**

- We would like to thank Dr Kawade for their positive feedback on the factorial trial extensions and their suggestions for adding to the guidance. However, this paper reports the methods/process employed to develop SPIRIT and CONSORT extensions for factorial trials. As mentioned in the manuscript, the extension items have been published, and therefore it is not possible to make additional changes at this time.

**Reviewer: 4: Dr. Ellen Elsman, Amsterdam UMC Location VUmc**

1. I therefore wonder if there will be any subsequent evaluation of item wording and how improvements or revisions are planned?

- We believe the approach taken provided an effective means to gather wider opinions on the general importance of the concepts to be included in the extension documents, and then refine the wording of these concepts with experts in writing, which is a highly specialised skill-set not afforded by all researchers. We have reflected on this in our discussion as both a strength and a limitation:

"Indeed, we spent time during the two-day meeting considering the detailed wording of specific items derived from the concepts. This approach enabled us to gain rich, nuanced information from international experts in writing (including editors of leading journals including JAMA and BMJ) which would not be possible in a Delphi survey."

"Research to support the validity of these extension items should include piloting with less experienced researchers, evaluating individual item comprehensibility."

2. Another concern is about the pilot testing. It seems that pilot testing was conducted with people who were very involved in the project and thus are very familiar with the meaning of reporting items. Wouldn't it be better to pilot test with people new to the reporting items, so that more information about the clarity of items can be obtained? Also, pilot testing is very briefly described, and would benefit from more information. For example, how was the checklist pilot tested? How many articles were used? What was the feedback? Was the E&E document pilot tested as well?

- We have provided further information in the Methods and reflected upon the limitations of our piloting approach in the Discussion:

"Three authors (AAM, BK, EJ) pilot tested the extensions by each considering a different completed factorial randomised trial and using the checklists to briefly outline key points in the study protocol and main results publication manuscript. This process enabled the authors to reflect on the clarity of the item wording."

"Finally, the piloting process used represents the study's fifth limitation. The checklist items were internally piloted by members of the immediate research team. Research to support the validity of these extension items should include piloting with less experienced researchers, evaluating individual item comprehensibility."

3. PPI in reporting guideline development is relatively new. There were 2 PPI representatives involved in the Delphi study. Was there role solely PPI representative, or did they had other job roles as well (e.g., research-focused roles)? If not, were you able to collect experiences from them, and were there any measures in place to make sure they were able to understand the process and content? In the discussion, you already reflect on the limitation that only two PPI members were involved, and this might be a good place to refer to guidelines on PPI in reporting guideline development: "A blueprint for patient and public involvement in the development of a reporting guideline for systematic reviews of outcome measurement instruments: PRISMA-COSMIN for OMIs 2024." Research involvement and engagement 10.1 (2024): 33.

- We recognise the importance of adequate PPI involvement and thank the reviewer for brining this paper to our attention. Due to a lengthy review process, this paper was not published during the time of submission, nor was it published during the study design/concept phase. We will be reflecting upon this paper going forward with future endeavours. The following has been added to the discussion:

"A second limitation of the RAFT study is that we only had two Patient and Public Involvement (PPI) participants. Participants were requested to select their main role in relation to factorial trials, and as such it is possible that they also undertook other research related roles. No additional feedback was requested from these PPI members regarding their inclusivity of the study design from a lay perspective. Whilst PPI is not commonly considered in the development of reporting guidelines, patients often do their own information seeking with regards to healthcare options (37-39) and therefore developing guidelines which promote the readability of this information to patients could be important. How best to involve PPI members who may lack the necessary experience in key research terminology is an important consideration for future research, and since conducing this study published guidance is now available (40)."

4. It is stated that factorial trial designs are often not reported completely. Which aspects in particular are often reported incompletely? Could you give some examples? This might help the reader better understand the reporting issues that contribute to limited applicability.

- We have provided further context in the Background:

"The use of a factorial design introduces additional methodological complexities, which are often inadequately understood and poorly reported (12-15). A review of 100 2x2 factorial trials published between January 2015 and March 2018 found a number of concerns around their design, analysis and reporting (12), including (i) providing no clear rationale for the factorial design, (ii) a lack of reporting of interactions, and (iii) using a preliminary test of interaction to choose the analysis method."

5. Please provide more information on the 6 additional consensus meeting participants. Who were they and how were they selected? Did they participate in the Delphi study as well? And where they all joining online?

- Further details have been added to the manuscript:

"A two-day hybrid meeting was held at the University of Nottingham. The 15 participants comprised the core RAFT team (5 attended in-person, 4 online) plus six leading experts in guideline developments and publishing as well as trial design (4 in-person, 2 online). The six experts all participated in the Delphi survey and had an international reputation for improving reporting standards, including authors of previous SPIRIT and CONSORT papers (31) and editors for leading journals (BMJ and JAMA). At the meeting, items which had reached consensus to include were discussed in terms of their utility in being included in the extension documents, and in terms of their specific wording in the final documents. Items which did not reach consensus were also evaluated for their importance to include in the final documents."

6. Items relating to the abstract were not evaluated in the Delphi survey but were chosen after completion of the main checklist. Why was this approach followed, how were items chosen and by whom?

- Further information on this process has been added:

"Items relating to the CONSORT extension for Abstracts (31) were not evaluated in the Delphi survey. Instead, these were chosen after the main checklist was completed in collaboration with the main author of the original CONSORT for Abstracts (31). This approach enabled items that were agreed for inclusion in the main extension document to be considered and combined with expert topic knowledge for abstract writing, preventing the generation of an overly burdensome list given the word limits applied to abstracts."

7. What strategies for dissemination of the RAFT guidelines are in place?

- This has been added to the results section of the workshop:

"Plans for dissemination included identifying target journals for the main checklist and accompanying Explanation and Elaboration document, highlighting the importance of publishing the methods for developing the extension checklist to ensure transparency of the process. Plans to conduct workshops/talks, including at the 2024 International Conference for Clinical Trials Methodology Conference (33) were also discussed with the aim of supporting researchers interested in factorial trials to conduct well reported trials at all stages from design (i.e., the protocol) to the results (i.e., the manuscript)."

8. Empirical studies quantify uncertainty a number of ways, but it is relevant to any scientific endeavor. How do you characterize uncertainty in the process of developing the RAFT guidelines? Obviously if you did the whole thing again (w/ or w/o different participants) you would expect some variability in the outcome. How is this characterized for this particular study?

- We have reflected upon issues relating to uncertainty and generalisability in the Discussion and Conclusion of the manuscript, please see extracts below:

"Our consensus threshold was clearly pre-defined, to ensure this was operationalised without uncertainty."

"Secondly, we involved key stakeholders from different job-roles (e.g., statisticians, trialists, editors); including potential end-users who may have different perspectives throughout the process to help ensure the extensions were applicable to a wide audience. Our targeted recruitment strategy resulted in over 80% of our final sample having experience of at least one factorial trial, ensuring topic-specific expertise. However, it is also advantageous that 20% had not been involved in such trials, to provide independent input on the face value of the proposed items."

"Thirdly, by conducting a scoping review of the relevant literature we were able to consider issues which widely affect the reporting of factorial trials at an international level and then evaluate the potential importance of these issues through a Delphi survey. Indeed, this process proved highly effective, as evidenced by the majority of original items being rated as important to include and the high degree of consensus reached in the Delphi survey, with only three survey items not reaching consensus after round 3."

"Fourthly, although a mixed-methods approach (including literature reviews and consensus-based methods), alongside substantial sample sizes, increases the generalisability and comprehensiveness of the proposed items it remains possible that other important concepts relating to a factorial design should be reported in a study protocol and manuscript. As such, we advise the SPIRIT and CONSORT extensions are viewed as a minimum set of items to include in the reporting process, as congruent with the original aim of the CONSORT and SPIRIT statements (1, 3)."

"By obtaining a larger sample of respondents we were able to gain wider representation of expert opinions to reach consensus on the final extension documents, helping to reduce content uncertainty and ensure the guidance is broadly applicable to factorial trial designs."

"By reporting the methodological stages involved in the developed of the RAFT guidelines this paper provides transparency for the process."

**Minor issues:**

1. Spell out RAFT in the abstract

- This has been added to background of the Abstract

2. “Funding was awarded from the Medical Research Council (MRC) Methodology Research Panel to support researcher time, purchase the Delphi software, host a consensus meeting. and support with dissemination.” > please remove the period in the middle of the sentence.

- This has been corrected

3. “First, our experienced Delphi panel members were provided the opportunity to suggest additional items in round of the Delphi survey.” > should read round 1?

- This has been corrected

4. “A second limitation of the RAFT study is that we only had two from Patient and Public Involvement (PPI) participants.” > please deleted from

- This has been corrected

---

## VERSION 2 - REVIEW

---

**Reviewer** 2

**Name** Jamrozik, Euzebiusz

**Affiliation** University of Oxford, Ethox and the Wellcome Centre for Ethics and Humanities

**Date** 13-Dec-2024

**COI**

---

Ready for publication from my perspective.

---

**Reviewer** 3

**Name** Kawade, Anand

**Affiliation** King Edward Memorial Hospital, Vadu Rural Heath Progrm

**Date** 13-Dec-2024

**COI**

---

Thanks for addressing the comments.

---

**Reviewer** 4

**Name** Elsman, Ellen

**Affiliation** Amsterdam UMC Location VUmc, Epidemiology & Data Science

**Date** **24-Dec-2024**

**COI**

---

Thank you for adequately addressing the comments of all reviewers.
